# Bacteriocins: Novel Solutions to Age Old Spore-Related Problems?

**DOI:** 10.3389/fmicb.2016.00461

**Published:** 2016-04-08

**Authors:** Kevin Egan, Des Field, Mary C. Rea, R. Paul Ross, Colin Hill, Paul D. Cotter

**Affiliations:** ^1^School of Microbiology, University College CorkCork, Ireland; ^2^Teagasc Food Research Centre, MooreparkFermoy, Ireland; ^3^APC Microbiome InstituteUniversity College Cork, Ireland; ^4^College of Science, Engineering and Food Science, University College CorkCork, Ireland

**Keywords:** antimicrobial peptide, bacteriocin, spore, *Bacillus*, *Clostridium*, food processing, LAB

## Abstract

Bacteriocins are ribosomally synthesized antimicrobial peptides produced by bacteria, which have the ability to kill or inhibit other bacteria. Many bacteriocins are produced by food grade lactic acid bacteria (LAB). Indeed, the prototypic bacteriocin, nisin, is produced by *Lactococcus lactis*, and is licensed in over 50 countries. With consumers becoming more concerned about the levels of chemical preservatives present in food, bacteriocins offer an alternative, more natural approach, while ensuring both food safety and product shelf life. Bacteriocins also show additive/synergistic effects when used in combination with other treatments, such as heating, high pressure, organic compounds, and as part of food packaging. These features are particularly attractive from the perspective of controlling sporeforming bacteria. Bacterial spores are common contaminants of food products, and their outgrowth may cause food spoilage or food-borne illness. They are of particular concern to the food industry due to their thermal and chemical resistance in their dormant state. However, when spores germinate they lose the majority of their resistance traits, making them susceptible to a variety of food processing treatments. Bacteriocins represent one potential treatment as they may inhibit spores in the post-germination/outgrowth phase of the spore cycle. Spore eradication and control in food is critical, as they are able to spoil and in certain cases compromise the safety of food by producing dangerous toxins. Thus, understanding the mechanisms by which bacteriocins exert their sporostatic/sporicidal activity against bacterial spores will ultimately facilitate their optimal use in food. This review will focus on the use of bacteriocins alone, or in combination with other innovative processing methods to control spores in food, the current knowledge and gaps therein with regard to bacteriocin-spore interactions and discuss future research approaches to enable spores to be more effectively targeted by bacteriocins in food settings.

## Introduction

Control and eradication of *Bacillus* and *Clostridium* spores is one of the most challenging aspects of microbial control faced by the modern food industry. Traditionally, spores have been controlled using extreme treatments such as high heat alone or in combination with chemical additives. However, modern consumers are more conscious than previous generations of the negative health effects associated with the consumption of certain chemical preservatives and of the significant effects of heat on the nutritional value and flavor of many foods. With ready-to-eat and minimally processed foods becoming a staple of the modern diet, the food industry is faced with an unprecedented challenge to provide food that is: (i) low in synthetic chemical additives, (ii) low in salt/sugar, (iii) nutritionally beneficial, and (iv) stable and safe, from a microbial perspective, over an extended period of time. As a result, the food industry is under pressure to employ innovative processing methods to meet consumer and regulatory demands. One potential innovation that has been intensively researched over the last number of decades, and is well positioned to provide a safe and effective alternative to existing processing technologies, involves the use of bacteriocins. This review will examine the efficacy of bacteriocins alone, and in combination with other processing technologies, to control spores in food.

## The bacterial spore

**Figure 1:**
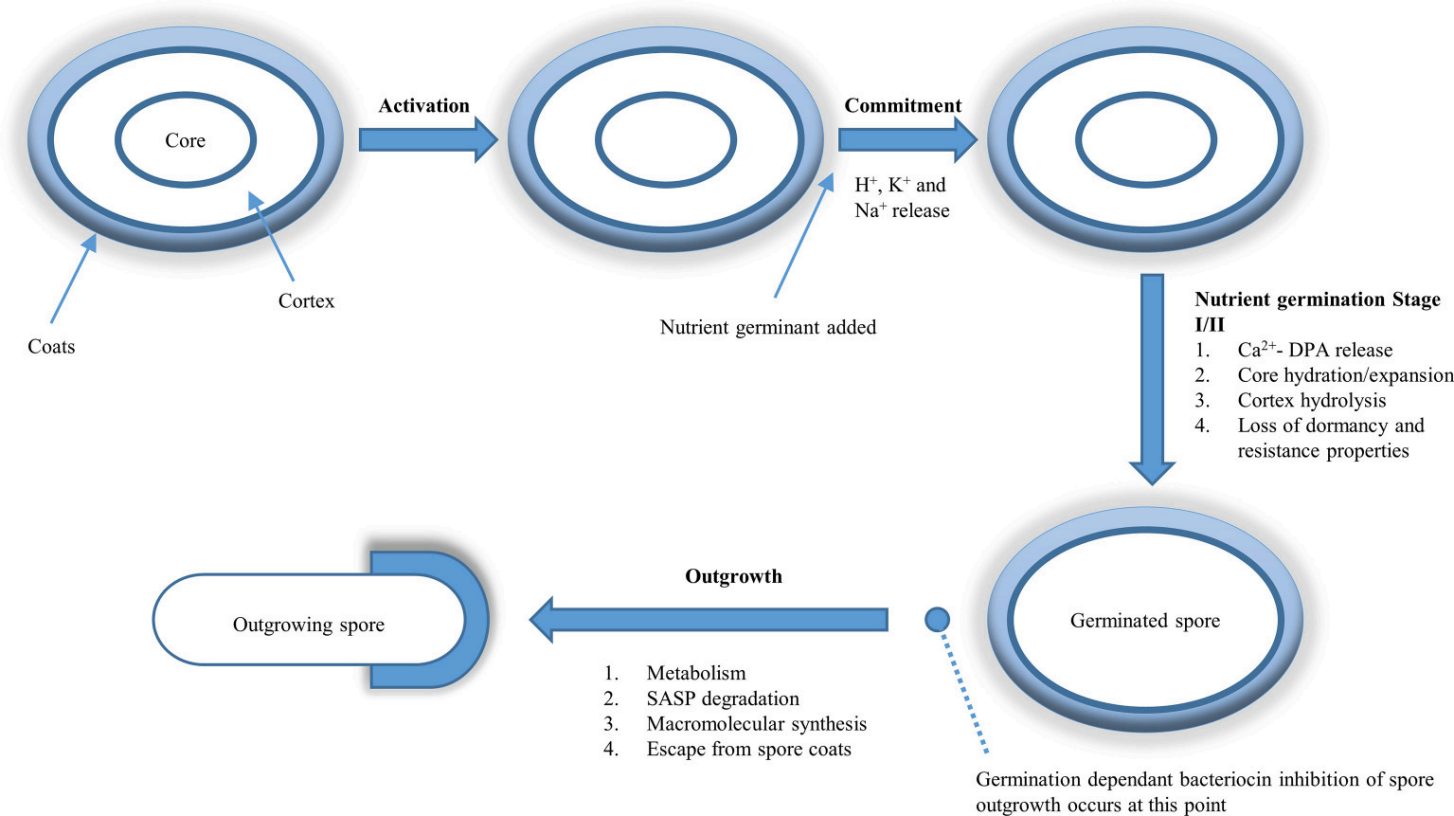
**Germination dependent inhibition of spore outgrowth by bacteriocins**. Dormant spores may germinate after being activated by a variety of means; most commonly sub-lethal heat being used. Heat is believed to activate the dormant spores by making the germinant receptors (GR) more accessible to nutrient germinants. Once the GR-nutrient binding occurs, the spore is now committed to germination even if the germinant is removed. Stage 1 of germination consists of H^+^, K^+^, and Na^+^ ion release followed by Ca^2+^-DPA release. This release of Ca^2+^-DPA triggers stage II of germination where the cortex is degraded, allowing the germ cell wall to expand and take up water. At the end of stage II the spore core is hydrated and has expanded along with the cortex. This rise in water content signals the end of stage II of germination and the beginning of the outgrowth phase. At this point bacteriocins that are not active against dormant spores become active, inhibit outgrowth and reduce viable counts from the germinated spore population. This figure is adapted from Setlow (2014a).

Metabolically dormant spores of Gram-positive *Clostridium* and *Bacillus* species are formed during sporulation. This sporulation process is typically a response to cellular nutrient starvation and involves a complex cascade of enzyme reactions. This process of sporulation has been extensively described over the last number of decades in the model spore former *B. subtilis* (see review by: Tan and Ramamurthi (2014). Spores consist of a core surrounded by a coat and/or endosporium. The spore core consists of DNA, enzymes, and dipicolinic acid (DPA). DPA plays a role in maintaining spore dormancy, providing resistance to DNA damaging substances and is usually bound to divalent cations such as Ca^2+^ at a 1:1 ratio in the core (Setlow, 2014b). The composition and structure of the metabolically inactive, dehydrated, spore confers resistance to changes in pH (Blocher and Busta, 1983), wet and dry heat, UV radiation, desiccation (Nicholson et al., 2000), and various toxic chemicals (Russell, 1990; Cortezzo and Setlow, 2005). A spore may be viable after extended periods of dormancy (Cano and Borucki, 1995), monitoring its environment for favorable growth conditions and when suitable, germination and outgrowth occur, ultimately resulting in a vegetative cell (Figure 1). Endospore-forming bacteria vary considerably with respect to genotype and phenotype and, with respect to phenotype, consist of aerobic, facultative anaerobic, and obligate anaerobic, psychrophilic, mesophilic, thermophilic, psychotropic and thermotolerant strains (see review by: Doyle et al., 2015). This phenotypic heterogeneity of spore-forming bacteria means that virtually all types of food are potential targets for spore contamination and spore outgrowth, with potentially severe consequences with respect to food quality and safety.

There are many pathways via which spores can gain access to the food chain. Food products are composed of multiple ingredients, potentially from different international origins, each contributing their own specific quantity and diversity of spores into the final formulation. Factors such as microbial ecology, farming practices, the local climate, hygiene of the processing facility and animal feeding practices determine the spore composition of an ingredient. Spores are also highly adhesive and may remain on the surfaces of equipment and contribute to problems long after their initial contamination of the facility. Reducing these initial spore loads is critical in avoiding problems downstream. However, it is important to note that spores are often selected for in food processing as their thermal resistance allows them to endure any heating steps (see review by: Carlin, 2011).

As early as 1956 (Stuy, 1956), the induction of spore germination was identified as a strategy that could facilitate spore eradication. When threshold levels of nutrients (such as amino acids, sugars, and nucleosides) are present, they bind to Ger complexes, located on the inner membrane of the spore. This strategy takes advantage of the loss of the resistance properties that a dormant spore possesses. It has been shown that once spores have germinated, they become more sensitive than dormant spores to: heat (Durban et al., 1970), X-Ray and UV radiation (Stuy, 1956; Munakata, 1974), and copper (Wheeldon et al., 2008). Interestingly the process of spore germination is not 100% efficient, due to the heterogeneity in germination rates among members of the spore population in response to a particular nutrient germinant. Previous studies have highlighted the specificity of germinant receptors (GRs): showing that GerA will respond to L-alanine and L-valine, while GerB and GerK will respond to a mixture of L-asparagine, D-glucose, D-fructose, and potassium ions (Moir et al., 1994; Atluri et al., 2006). The binding of the nutrients to their appropriate GRs results in the irreversible commitment of the spore to germination.

Commonly spores termed superdormant have been isolated from populations of *B. subtilis* following saturation with nutrient germinant. This super dormancy is attributed to the lag in initiation of the rapid loss of Ca^2+^-DPA stage in spore germination. Following the initiation of rapid loss of Ca^2+^-DPA from its core, the spore is no longer superdormant and its germination will proceed in a similar manner as dormant spores (Figure 1). This superdormancy may be an issue for antimicrobials (e.g. nisin) whose effect is only exhibited on those spores that have reached the end of stage II of germination (Figure 1; Chen et al., 2014). Superdormant spores may, however germinate, in response to an alternative germinant that utilizes an alternative GR. A different strategy, which can be used to increase germination of super dormant spores, is by using higher heat activation temperatures than is required for those non-superdormant spores (Ghosh et al., 2009). Treatment of spores with sublethal heat (also called heat activation) has been shown to increase the rate of germination of a number of spore species. Luu et al. (2015) suggested that although the main target of heat activation is the spore's GRs, this may only be indirect and that the sublethal heat is having a more direct effect on the inner membrane of the spore in which the GRs are situated, ultimately resulting in increased spore germination. Therefore decisive triggering of the spore germination process, will allow food processors to render spores sensitive to a variety of inactivation methods that are ineffective against highly resistant dormant spores.

## Bacteriocins

Bacteriocins are a class of ribosomally synthesized antimicrobial peptides (AMPs) produced by bacteria. These small and naturally produced peptides can kill other bacteria, which are closely (narrow spectrum) or distantly (broad spectrum) related to the producing bacteria (Cotter et al., 2005). It is hypothesized that the production of bacteriocins is a strategy to control competing bacteria in the hunt for nutrients and space in an environmental niche. Therefore, it is not surprising that it has been estimated that many bacteria produce at least one bacteriocin (Riley and Wertz, 2002), which may help them to influence the surrounding population dynamics. Although many bacteriocin-producing bacteria in the biosphere have been investigated, it is still the case that there remain many are that are still to be discovered (Yang et al., 2014). Indeed, bioinformatic mining of publically available genomes, along with other rapid techniques, are beginning to bridge this gap in initial discovery, by overcoming the previous dependence on the expensive, time consuming, culture-dependent nature of bacteriocin discovery and purification (Sandiford, 2015). BAGEL3 (**BA**cteriocin **G**enome mining too**L**) (van Heel et al., 2013) and antiSMASH 3.0 (**anti**biotics and **S**econdary **M**etabolite **A**nalysis **S**hell) (Weber et al., 2015) are examples of web based genome mining tools that detect putative bacteriocin biosynthetic gene clusters. Liquid chromatography/mass spectrometry has also been used to rapidly detect bacteriocins in as little as 25 μl of culture supernatant and is sensitive enough to distinguish between variants of the same bacteriocin e.g., nisins A, Z, and Q (Zendo et al., 2008). High throughput, culture-based screens can also be valuable (Rea et al., 2010).

### Bacteriocins from the LAB are suitable for food preservation

Although there are many Gram-negative and Gram-positive microorganisms which produce bacteriocins, those produced by the lactic acid bacteria (LAB) are of particular interest to the food industry. Many of these bacteria already play a crucial role in a variety of food fermentations by converting lactose to lactic acid, as well as producing a variety of additional antimicrobial molecules such as other organic acids, diacetyl, acetoin, hydrogen peroxide, antifungal peptides, and bacteriocins. The best known LAB genera are *Lactococcus, Streptococcus, Lactobacillus, Pediococcus*, and *Enterococcus*, though a number of other, generally regarded as more peripheral and less frequently applied from an industrial perspective, genera also exist. LAB offer several key properties which make their bacteriocins highly desirable for use in food: (i) the LAB are Generally Regarded As Safe (GRAS) and there are perceived by the public as having health promoting features, (ii) their bacteriocins are sensitive to digestive proteases such as pancreatin complex, trypsin and chymotrypsin, and thus don't impact negatively on the gut microbiota, (iii) they are non-toxic to eukaryotic cells (iv) they are often active across a range of pH values and are, in many cases, not temperature sensitive (Table 1), (v) they are gene encoded and therefore highly amenable to genetic manipulation where desired (Field et al., 2015), (vi) not all of the bacteriocins produced by the LAB have similar/the same mode of action, and (vii) they are active against a range of food pathogenic and spoilage bacteria.

**Table 1:** **Bacteriocins that are active against vegetative cells of Gram-positive spore-forming bacteria**.

**Bacteriocin**	**Class**	**Producer**	**Size (Da)**	**Spectrum**	**Heat stability**	**Active pH**	**Sensitive Spore-formers**	**References**
Acidocin LCHV	IId	*Lactobacillus acidophilus* n.v. Er 317/402 strain narine	1158.2^c^	Broad	Heat stable	3–8	*B. cereus* *B. subtilis*	Mkrtchyan et al., 2010
Acidocin LF221A Acidocin LF221B	IIb	*Lactobacillus gasseri* LF221	3500-5000^a^	Broad	Heat stable	2–9	*B. cereus* *C. sporogenes* *C. tyrobutyricum*	Bogovic-Matijasić et al., 1998
Bac217	IId	*Lactobacillus paracasei* subsp. *paracasei* BGBUK2-16	7000^a^	Broad	Heat stable	3–12	*B. cereus* *B. fragilis* *B. subtilis*	Lozo et al., 2004
BacC1	ND	*Enterococcus faecium* C1	10,000^a^	Broad	Heat stable	2–6	*B. cereus*	Goh and Philip, 2015
Bacteriocin L-1077	IIa	*Lactobacillus salivarius* 1077	3454	Broad	ND	ND	*C. perfringens*	Svetoch et al., 2011
Bifidocin B	IIb	*Bifidobacterium bifidum* NFBC 1454	4432.9^c^	Narrow	Heat stable	2–10	*B. cereus*	Yildirim and Johnson, 1998b; Yildirim et al., 1999
Bificin C6165	ND	*Bifidobacterium animalis* subsp. animalis CICC 6165	3395.1^c^	Narrow	Moderate	3.5–6.5	*A. acidoterrestris*	Pei et al., 2013
Brevicin 925A	IId	*Lactobacillus brevis* 925A	ND	Narrow	Heat resistant	ND	*B. coagulans*	Wada et al., 2009
Divergicin 750	IId	*Carnobacterium divergens* 750	3447.7	Broad	ND	ND	*C. perfringens*	Holck et al., 1996
Duranicin TW-49M	IId	*Enterococcus durans* Q 49	5227.8^c^	Narrow	Moderate	2–10	*B. coagulans* *B. circulans* *B. subtilis* *G. stearothermophilus*	Hu et al., 2008
Enterocin 7A/7B	IId	*Enterococcus faecalis* 710C	7A 5200.8^c^ 7B 5206.65^c^	Broad	ND	ND	*C. butyricum* *C. botulinum* *C. perfringens* *C. sporogenes*	Liu et al., 2011
Enterocin A	IIa	*Enterococcus faecium* CTC492, *Enterococcus faecium* T136	3829^c^	Broad	Heat stable	2–10	*B. coagulans* *B. subtilis* *C. sporogenes* *C. tyrobutyricum*	Aymerich et al., 1996; Casaus et al., 1997; Hu et al., 2010, 2014
Enterocin AS-48	IIc	*Enterococcus faecalis* A-48-32	7140^c^	Broad	Heat stable	ND	*Alicyclobacillus* spp. B. cereus B. coagulans B. licheniformis B. subtilis C. perfringens C. sporogenes C. tetani G. stearothermophilus Paenibacillus spp.	Lucas et al., 2006b; Burgos et al., 2014
Enterocin B	IId	*Enterococcus faecium* T136 Ent	5463^c^	Broad	Heat stable	ND	*B. coagulans* *B. subtilis* *C. sporogenes* *C. tyrobutyricum*	Casaus et al., 1997; Hu et al., 2010
Enterocin EJ97	IId	*Enterococcus faecalis* EJ97	5340^c^	Broad	Heat stable	2–9.5	*B. circulans* *B. coagulans* *B. macrolides* *B. megaterium* *B. moroccanus* *B. subtilis* *G. stearothermophilus* *Paenibacillus macerans*	Gálvez et al., 1998; Garcia et al., 2004
Enterocin L50	IIb	*Enterococcus faecium* L50	A: 5190^c^ B: 5178^c^	Broad	Heat stable	2–11	*B. cereus* *B. subtilis*	Cintas et al., 1995; Basanta et al., 2010
Enterocin IT	IId	*Enterococcus faecium* IT62	6390^c^	Narrow	ND	ND	*B. subtilis*	Izquierdo et al., 2008
Enterocin MR10	IIb	*Enterococcus faecalis* MRR10-3	A: 5201.6^b^ B: 5207.5^b^	Broad	Heat stable	4.6–9	*B. cereus* *B. licheniformis*	Martín-Platero et al., 2006
Enterocin NKR-5-3B	IIc	*Enterococcus faecium* NKR-5-3	6316.42^c^	Broad	Heat stable	2–10	*B. circulans* *B. coagulans* *B. subtilis*	Himeno et al., 2015
Enterocin RM6	IId	*Enterococcus faecalis* OSY-RM6	7145^c^	Broad	ND	ND	*B. cereus*	Huang et al., 2013
Enterocin P	IId	*Enterococcus faecium* P13	4493^b^	Broad	Heat stable	2–11	*B. cereus* *C. botulinum* *C. perfringens* *C. sporogenes* *C. tyrobutyricum*	Cintas et al., 1997
Enterocin SE-K4	IIa	*Enterococcus faecalis* K-4	5356.2^c^	Narrow	Heat stable	3–11	*B. subtilis* *C. beijerinckii*	Eguchi et al., 2001
Gassericin A	IIc	*Lactobacillus gasseri* LA 39	3800^a^	Broad	Heat stable	2–12	*B. cereus*	Nakamura et al., 2013
Gassericin KT7	ND	*Lactobacillus gasseri* KT7	ND	Broad	Heat stable	2.5–9	*B. cereus* *B. subtilis* *C. botulinum* *C. perfringens*	Zhu et al., 2000
Garvieacin Q	IId	*Lactococcus garvieae* BCC 43578	5339^c^	Broad	Heat stable	2–8	*B. coagulans*	Tosukhowong et al., 2012
Lacticin 3147	I	*Lactococcus lactis* subsp. *lactis* DPC3147	ltnA1: 3305^c^ ltnA2: 2847^c^	Broad	Heat stable	5–9	*B. cereus* *B. subtilis* *C. sporogenes* *C. tyrobutyricum*	McAuliffe et al., 1998; Martinez-Cuesta et al., 2010; Iancu et al., 2012
Lacticin 481	I	*Lactococcus lactis* subsp. *lactis* CNRZ 481	2901^c^	Narrow	Heat stable	ND	*C. tyrobutyricum*	Piard et al., 1990, 1993
Lacticin LC14	ND	*Lactococcus lactis* BMG6. 14	3333.7^c^	Broad	Heat stable	2–10	*B. cereus* *B. thuringiensis*	Lasta et al., 2012
Lacticin Q	IId	*Lactococcus lactis* QU 5	5926.5^c^	Broad	Heat stable	2–10	*B. cereus* *B. circulans* *B. coagulans*	Fujita et al., 2007
Lacticin Z	IId	*Lactococcus lactis* QU 14	5968.9^c^	Broad	Heat stable	2–10	*B. subtilis* *B. circulans* *B. coagulans*	Iwatani et al., 2007
Lactococcin BZ	ND	*Lactococcus lactis* subsp. *lactis*	5500^a^	Broad	Heat stable	2–7	*B. cereus* *B. subtilis*	Sahingil et al., 2011
Lactococcin R	ND	*Lactococcus cremoris* subsp. *cremoris* R	2500^a^	Broad	Heat stable	2–9	*B. cereus* *B. subtilis* *C. perfringens* *C. sporogenes*	Yildirim and Johnson, 1998a
Leucocin H	IIb	*Leuconostoc* MF215B	ND	Broad	ND	ND	*B. cereus* *C. perfringens*	Blom et al., 1999
Leucocyclicin Q	IIc	*Leuconostoc mesenteroides* TK41401	6115.59^c^	Broad	ND	ND	*B. cereus* *B. coagulans* *B. subtilis*	Masuda et al., 2011
Lactocyclin Q	IIc	*Lactococcus* sp. strain QU 12	6062^c^	Broad	Heat stable	3–9	*B. cereus* *B. coagulans* *B. subtilis*	Sawa et al., 2009; Masuda et al., 2011
Mesentericin ST99	ND	*Leuconostoc mesenteroides* ST99	ND	Broad	Heat stable	2–12	*B. subtilis*	Todorov and Dicks, 2004
Macedocin	I	*Streptococcus macedonicus*	2795^c^	Broad	Heat stable	4–9	*B. cereus* *B. subtilis* *C. sporogenes* *C. tyrobutyricum*	Georgalaki et al., 2002
Macedovicin	I	*Streptococcus macedonicus* ACA-DC 198	3428.8^c^	Broad	ND	ND	*B. licheniformis* *C. sporogenes* *C. tyrobutiricum*	Georgalaki et al., 2013
Nisin	I	*Lactococcus lactis* subsp. *lactis*	3353.53^c^	Broad	Heat stable	2–6	*A. acidoterrerstris* *B. anthracis* *B. amyloliquefaciens* *B. cereus* *B. coagulans* *B. fliexus* *B. licheniformis* *B. pumilus* *B. sporothemodurans* *C. beigerinckii* *C. butyricum* *C. perfringens* *C. sporogenes* *C. tyrobutyricum* *Paenbacillus jamilae*	Meghrous et al., 1999; Pirttijärvi et al., 2001; Wijnker et al., 2011; Hofstetter et al., 2013; Oshima et al., 2014; Aouadhi et al., 2015
Nisin Z	I	*Lactococcus lactis* NIZO 22186	3330.93	Broad	Heat stable	2–6	*B. cereus* *B. pumilus* *B. subtilis* *C. butyricum* *C. perfringens* *C. sporogenes* *C. tyrobutyricum*	Rollema et al., 1995; Meghrous et al., 1999; Noonpakdee et al., 2003; Park et al., 2003; Rilla et al., 2003; Rumjuankiat et al., 2015
Nisin Q	I	*Lactococcus lactis* 61-14	3327.5	Broad	Heat stable	ND	*B. circulans* *B. coagulans* *B. subtilis*	Zendo et al., 2003
Pediocin A	IIa	*Pediococcus pentosaceus* FBB61	80,000^a^	Broad	Heat sensitive	ND	*B. cereus* *C. sporogenes* *C. tyrobutyricum*	Piva and Headon, 1994
Pediocin AcH/PA-1	IIa	*Pediococcus acidilactici* PAC 1.0	4624^c^	Broad	Heat stable	2–10	*B. cereus* *C. butyricum* *C. perfringens* *C. sporogenes* *C. tyrobutyricum*	Marugg et al., 1992; Meghrous et al., 1999; Rodríguez et al., 2002; Nieto-Lozano et al., 2010
Pediocin AcM	IIa	*Pediococcus acidilactici* M	4618^c^	Broad	Heat stable	1–12	*B. cereus* *B. coagulans* *C. perfringens*	Elegado et al., 1997
Pediocin L50	IId	*Pediococcus acidilactici* L50	5250^c^	Broad	Heat stable	2–11	*B. cereus* *C. botulinum* *C. perfringens* *C. sporogenes* *C. tyrobutyricum*	Cintas et al., 1995
Pentocin TV35b	ND	*Lactobacillus pentosus* TV35b	3930	Broad	Heat stable	1–10	*C. sporogenes* *C. tyrobutyricum*	Okkers et al., 1999
Plantaricin 163	IId	*Lactobacillus plantarum* 163	3553.2	Broad	Heat stable	2–10	*B. cereus*	Hu et al., 2013
Plantaricin 423	IIa	*Lactobacillus plantarum* 423 *Lactobacillus plantarum* LMG P-26358	3932^c^	Narrow	Heat stable	1–10	*B. cereus* *C. sporogenes*	van Reenen et al., 1998; Mills et al., 2011
Plantaricin C	Ì	*Lactobacillus plantarum* LL441	2880.3^c^	Broad	Heat stable	< 8	*B. subtilis* *C. sporogenes* *C. tyrobutyricum*	Gonzalez et al., 1994
Plantaricin KL-1Y	IId	*Lactobacillus plantarum* KL-1	3497.97^c^	Broad	Heat stable	2–12	*B. cereus* *B. coagulans* *B. subtilis*	Rumjuankiat et al., 2015
Plantaricin LP84	ND	*Lactobacillus plantarum* NCIM 2084	1000 - 5000^a^	Broad	Heat stable	ND	*B. cereus* *B. licheniformis* *B. subtilis*	Suma et al., 1998
Plantaricin PZJ5	IId	*Lactobacillus plantarum* ZJ5	2572.9^c^	Broad	Heat stable	2–6	*B. subtilis*	Song et al., 2014
Plantaricin S	IIb	*Lactobacillus plantarum* LPC010	α 2904^c^ β 2873^c^	Broad	Heat stable	3–7	*C. tyrobutyricum*	Soliman et al., 2011
Plantaricin ST31	ND	*Lactobacillus plantarum* ST31	2755^c^	Broad	Heat stable	3–8	*B. subtilis*	Todorov et al., 1999
Plantaricin TF711	ND	*Lactobacillus plantarum* TF711	2500^a^	Broad	Heat stable	1–9	*B. cereus* *C. sporogenes*	Hernández et al., 2005; González and Zárate, 2015
Plantaracin UG1	ND	*Lactobacillus plantarum* UG1	3000-10,000^a^	Narrow	Heat stable	3.5–8	*B. cereus* *C. perfringens* *C. sporogenes*	Enan et al., 1996
Plantaricin ZJ008	ND	*Lactobacillus plantarum* ZJ008	1334.77	Broad	Heat stable	2–8	*B. subtilis*	Zhu et al., 2014
Salivaricin D	I	*Streptococcus salivarius* 5M6c	3467.55	Broad	Heat stable	ND	*B. subtils* *C. butyricum* *C. bifermentans*	Birri et al., 2012
Thermophilin 1277	I	*Streptococcus thermophilus* SBTI1277	3700^a^	Broad	Heat stable	3–10	*B. cereus* *C. butyricum* *C. sporogenes* *C. tyrobutyricum*	Kabuki et al., 2007
Themophilin 13	IIb	*Streptococcus thermophilus* SFi13	5776^c^	Broad	ND	ND	*B. cereus* *B. subtilis* *C. botulinum* *C. tyrobutyricum*	Marciset et al., 1997
Thermophilin T	ND	*Streptococcus thermophilus* ACA-DC 0040	2500^a^	Narrow	Heat stable	1–12	*C. sporogenes* *C. tyrobutyricum*	Aktypis et al., 1998
VJ13B	IIa	*Pediococcus pentosaceus* VJ13	4000^a^	Broad	Moderate	2–8	*B. cereus* *B. subtilis* *C. perfringens* *C. sporogenes*	Vidhyasagar and Jeevaratnam, 2013
Weissellicin Y	IId	*Weisella hellenica Q13*	4925^c^	Broad	Heat stable	3–11	*B. cereus* *B. circulans* *B. subtilis* *B. coagulans*	Masuda et al., 2012
Weissellicin M	IId	*Weisella hellenica Q13*	4968^c^	Broad	Moderate	3–11	*B. cereus* *B. circulans* *B. coagulans* *B. subtilis*	Masuda et al., 2012

a *Mass estimated using SDS-PAGE*.

b *Mass calculated based on amino acid sequence*.

c *Mass obtained using mass spectrometry*.

*ND, Not determined*.

This review will focus solely on bacteriocins produced by the LAB because these bacteriocins possess the greatest promise with respect to use in the food industry.

### Classification of bacteriocins produced by the LAB

LAB bacteriocins may be classified into two separate classes based on their modification status: Modified (class I), and minimally modified or cyclic (class II; Rea et al., 2011; Cotter et al., 2013).

Class I are comprised of all peptides that undergo post-translational modification during biosynthesis and include the subclass of lantibiotics among others. While several other subclasses within class I have been described (Arnison et al., 2013; Cotter et al., 2013), this review will focus mainly on those with relevance to the food industry. The commercially important bacteriocin nisin is produced by *L. lactis* and is the prototypical member of the class I lantibiotics. Nisin is currently used in over 50 countries to improve food safety and extend shelf life. Other important members of this class include: the two peptide lantibiotic lacticin 3147 produced by *L. lactis* DPC 3147 (Suda et al., 2012), subtilin produced by *Bacillus subtilis* ATCC 6633 (Lee and Kim, 2011), and lacticin 481 produced by *L. lactis* CNRZ 481 (Piard et al., 1993). Lantibiotics undergo extensive post-translational modifications, resulting in the presence of unusual amino acids such as lanthionine, β**-**methyllanthionine, dehydrobutyrine, and dehydroalanine. Covalent bonds are formed between these non-standard residues, resulting in internal rings which are important for its potent activity (Rink et al., 2007).

Class II bacteriocins are < 10 kDa, heat stable and non-modified that can be further subdivided into four subgroups: IIa pediocin like, IIb two peptide bacteriocins, IIc cyclic bacteriocins, and IId single linear non-pediocin bacteriocins. Members of class IIa are *Listeria*-active peptides which contain a conserved amino acid consensus sequence across all members of this group: Y-G-N-G-V-X_1_-C-X_2_-K/N-X_3_-X_4_-C (where X is any amino acid) (Cui et al., 2012). This consensus sequence is often referred to as the “pediocin box” and is present at the N-terminal region of the class IIa bacteriocins. Class IIb bacteriocins are unmodified two peptide bacteriocins, which interact to give full activity; having little or no activity in isolation. Class IIc bacteriocins are covalently linked from their N to C termini during post-translational modification resulting in a circular backbone. Class IId are a heterogeneous group, made up of bacteriocins which are linear, do not contain a pediocin box and do not require another peptide for full activity.

### Using bacteriocins produced by enterococcus in food

The bacteriocins produced by *Enterococcus* species are diverse, both in terms of their classification and inhibitory spectrum (Table 1). While most LAB are GRAS, and thus their associated bacteriocins can be considered for food applications, the status of enterococci is less clear. Indeed, many strains are clearly not food grade. Although *Enterococcus* species have been used as artisanal cultures in a variety of foods, their suitability for use in food is questionable as they have been sometimes associated with pathogenicity. Indeed, cases of urinary tract infections, bacteremia and endocarditis have been associated with *Enterococcus* species (Franz et al., 1999; Kayser, 2003). De Vuyst et al. (2003) suggested that *Enterococcus* species could be safely used in food if virulence genes are absent (cytolysin, vancomycin resistance, etc.). However, in a review by Franz et al. (2011), the ability of *Enterococcus* to acquire virulence and antibiotic resistance genes on mobile genetic elements was identified as a significant barrier to their use in food. Recently, Jaouani et al. (2015) examined the safety of previously identified bacteriocinogenic enterococci, by examining the presence of virulence and antibiotic resistance genes. Using these criteria, it was concluded that 22/55 of the strains tested were safe for use in food. Ultimately, *Enterococcus* are an important reservoir for bacteriocin discovery and therefore developing a comprehensive set of guidelines/considerations for their safe use would be highly valuable when considering their suitability for use in food.

### Bacteriocin mode of action against vegetative cells

Mechanistically, bacteriocin molecules produced by the LAB act by one, or both, of two different mechanisms: (i) inhibition of cell wall biosynthesis, and (ii) pore formation.

At the cell envelope, lipid II plays a key role in the synthesis of peptidoglycan as it transports cell wall subunits across the bacterial cytoplasmic membrane. Lipid II delivers its peptidoglycan subunit cargo from the cytosol to an exterior multi-enzyme complex which is responsible for polymerization of that subunit into the peptidoglycan cell wall. The halting of cell wall biosynthesis by sequestering lipid II is a strategy employed by a number of antimicrobial compounds which results in cell death (see review by: Oppedijk et al., 2015). The important clinical antibiotic vancomycin also targets lipid II, though its lipid II binding site is distinctly different to the lantibiotic nisin. The alternative binding site for nisin results in the ability of nisin to kill bacterial cells which are resistant to vancomycin (Gut et al., 2011). Other bacteriocins that exert their bactericidal mechanism of action by inhibition of cell wall biosynthesis are mersacidin, which inhibits transglycosylation (Brötz et al., 1995), and lactococcin 972 which targets septum biosynthesis via lipid II (Martínez et al., 2008). While lipid II is an important receptor for certain bacteriocins, there are however other receptors to which bacteriocins bind on the Gram-positive cell such as: the mannose PTS system, the maltose ABC transporter, Zn-dependent metallopeptidase, and undecaprenyl pyrophosphatase phosphatase (see review by: Cotter, 2014). Indeed these bacteriocin-receptor complexes play an important role in specifying a bacteriocins spectrum of activity. The outer cell membrane of Gram-negative bacteria provides an effective barrier to bacteriocins from binding their lipid II targets. However, Gram-negative bacteria can be sensitized toward bacteriocins if treated with agents or chemicals that destabilize the outer cell membrane (such as sodium phosphate buffer or EDTA).

Bacteriocins may also kill or damage cells by pore formation in the cell membrane. This pore formation is achieved by insertion of the bacteriocin into the cell membrane, forming a membrane pore. This pore results in depolarization of the membrane potential and diffusion of low molecular cytosolic compounds out of the cell; ultimately rendering the bacterial cell non-viable. Enterocin AS-48 is predicted to form aggregates which insert into the bacterial membrane, resulting in accumulation of positive charge along the cell surface, destabilizing the membrane potential, leading to pore formation and cellular leakage. Other bacteriocins that form pores include: streptococcin SA-FF22, lacticin F, and lactococcin A (Héchard and Sahl, 2002).

There are a number of members of the bacteriocins that exhibit dual modes of antimicrobial action by both: forming pores and inhibiting cell wall biosynthesis. The ability of such bacteriocins to act through two mechanisms of action can prevent the development of bacteriocin resistance. Moreover, it is worth noting that microorganisms that are resistant to antibiotics generally do not display cross-resistance to bacteriocins (Jordan et al., 2014). Nisin (Wiedemann et al., 2001), pediocin PA-1 (Diep et al., 2007), lacticin 3147 (Wiedemann et al., 2006), epidermin (Götz et al., 2014), and gallidermin (Götz et al., 2014) are examples of bacteriocins that display a dual mode of action, making their activity particularly potent against their targets.

## Bacteriocin-spore interactions

In comparison to the vast knowledge available with respect to bacteriocin interactions with vegetative cells, it is safe to say that there is considerably less known about bacteriocin/spore interactions. However, a small number of bacteriocins (Table 2) for which activity against a variety of bacterial spores has been demonstrated. Phase contrast microscopy can be utilized to determine at what stage in the spore cycle (Figure 1) the bacteriocin exhibits its anti-spore activity by combining the bacteriocins with dormant (phase bright) and germinated (phase dark) spores. Spore viability can then be examined following the treatment with bacteriocin to determine the bacteriocins effect on the spore. Two outcomes may ensue: the bacteriocin (i) does not require germination and will be sporicidal against dormant spores, or (ii) will be sporostatic to dormant and germinated spores but requires germination to inhibit spore outgrowth. Bacteriocins can also affect the germination rate of the spore, which can be examined by measuring the drop in absorbance (OD_600nm_) of a dormant spore suspension as it transitions to a germinated spore suspension over a time period. These outcomes are however heterogeneous (Table 3), with differences occurring at species level where the same bacteriocin was used, and will be further discussed below.

**Table 2:** **Bacteriocins that display inhibitory action against bacterial spores**.

**Bacteriocin**	**Sensitive spores**	**References**
Nisin	*A. acidoterrestris, B. amyloliquefaciens B. anthracis, B. licheniformis, B. sporothermodurans, B. subtilis, B. cereus, G. stearothermophilus, C. perfringens, C. sporogenes, C. botulinum, C. difficile, C. beijerinckii*	Komitopoulou et al., 1999; Mansour et al., 1999; Wandling et al., 1999; Pol et al., 2000; Black et al., 2008; Gut et al., 2008; Udompijitkul et al., 2012; Hofstetter et al., 2013; Nerandzic and Donskey, 2013; Aouadhi et al., 2015
Enterocin AS-48	*A. acidoterrestris, B. cereus, B. licheniformis, G. stearothermophilus*	Abriouel et al., 2002; Lucas et al., 2006
Bificin C6165	*A. acidoterrestris*	Pei et al., 2014
Lacticin 3147	*C. tyrobutyricum*	Martinez-Cuesta et al., 2010
Plantaricin TF711	*C. sporogenes*	González and Zárate, 2015
Thurincin H	*B. cereus*	Wang et al., 2014

**Table 3:** **Bacteriocin mode of action against bacterial spores is heterogeneous**.

**Bacteriocin**	**Spore**	**Effect on germination rate**	**Effect on dormant spores**	**Requires germination to be active**	**Other remarks**	**References**
Nisin	*B. anthracis*	None	None	Yes	Lipid II becomes available for nisin to bind following germination, followed by pore formation in the outgrowing spore.	Gut et al., 2008, 2011
	*B. sporothermodurans*	Decreased rate	None	Yes		Aouadhi et al., 2015
	*B. licheniformis*	None	None	Yes		Mansour et al., 1999
	*C. butyricum*	ND	None	Yes		Ramseier, 1960
	*C. botulinum*	Increases rate	None	Yes		Mazzotta and Montville, 1999
	*C. difficile*	None	None	Yes		Nerandzic and Donskey, 2013
	*C. perfringens*	None	None	Yes		Udompijitkul et al., 2012
Enterocin AS-48	*A. acidoterrestris*	ND	Sporicidal	No		Grande et al., 2005
	*B. cereus*	None	None	Yes		Abriouel et al., 2002
	*B. coagulans*	ND	None	Yes		Lucas et al., 2006
	*B. licheniformis*	ND	None	Yes		Grande et al., 2006a
	*G. stearothermophilus*	ND	None	Yes		Viedma et al., 2009
Thurincin H	*B. cereus*	None	None	Yes		Wang et al., 2014

*ND, Not determined*.

### Nisin

Previous studies have shown that for *B. anthracis* (Gut et al., 2008, 2011), *B. licheniformis* (Mansour et al., 1999), *C. difficile* (Nerandzic and Donskey, 2013), and *C. perfringens* (Udompijitkul et al., 2012), nisin had no impact on the process of germination, as it neither initiated, inhibited, or altered the rate of germination, as examined on the basis of spore refractility, with or without nisin. Conversely, the presence of 25 μg/ml of nisin has been shown to have a progerminant activity for *C. botulinum* spores, as when it was present in the germination medium, the germination rate was doubled. However, the presence of nisin (125 μg/ml) has been shown to decrease the germination rate of *B. sporothermodurans* spores (Aouadhi et al., 2015).

With respect to anti-*B. anthracis* activity, it has been reported that nisin exerts its inhibitory effect after germination initiation, where nisin binds lipid II in the germinating spore and prevents it from becoming metabolically active by interfering with the establishment of a membrane potential and oxidative metabolism. Germination initiation is required for this lipid II binding to occur, as nisin is unable to associate with the dormant spore due to the absence of lipid II on the exterior of the spore (Gut et al., 2011). When investigating the effects of nisin on *C. perfringens* spores, it was observed that, as for studies involving *B. anthracis* and *C. butyricum*, nisin exhibited its inhibitory action during the stage of spore outgrowth (Udompijitkul et al., 2012). Using a truncated nisin derivative consisting of rings A, B and C (which could bind lipid II but not form pores) and fluorescently labeled unmodified nisin, it was shown that lipid II binding alone was insufficient to inhibit spore outgrowth. This was further investigated using the double mutants N20P/M21P and M21P/K22P, which were unable to form pores, but could bind lipid II. These nisin mutants were again shown to be unable to inhibit spore outgrowth. Through the use of the double mutant and truncated nisin, it is clear that pore formation is the essential mechanism by which nisin inhibits spore outgrowth while lipid II is the target for nisin (acting as a receptor for nisin) to inhibit outgrowth in the germinating spore (Gut et al., 2008, 2011). While it has been shown that truncated nisin consisting of rings A, B, and C does not inhibit spore outgrowth in *B. anthracis*, it has been reported elsewhere that this peptide does inhibit outgrowth of *B. subtilis* (Rink et al., 2007). While the mechanisms underlying these differing results have not yet been completely elucidated, some possible explanations given were (i) differences in outgrowth measurement methods and (ii) potential spore structure variations (Gut et al., 2011). Nisin however displays sporicidal activity against dormant *B. sporothermodurans* (Aouadhi et al., 2013), in contrast with the sporostatic activity against other targets described above.

The ability of microorganisms to develop resistance mechanisms to bacteriocins is a concern that could impede their widespread use in food (see review by: Draper et al., 2015). Nisin resistance has been reported for toxigenic spores of *C. botulinum* which had the ability to germinate and grow in levels of nisin that reduced levels of sensitive germinating spores by 7–8 logs_10_/ml (Mazzotta and Montville, 1999). The exact mechanism by which these spores exhibited nisin resistance is unknown but, interestingly it has been noted that nisin resistant strains have an altered fatty acid composition, which is consistent with a more rigid membrane. It has also been observed that nisin resistant strains of *C. botulinum* display cross-resistance to class II bacteriocins (Mazzotta et al., 1997).

### Enterocin AS-48

Enterocin AS-48 produced by *Enterococcus faecalis* A-48-32 is a class IIc cyclic bacteriocin that is active against a number of *Bacillus* and *Clostridium* sp. (Table 1). Unlike nisin, the exact molecular mechanism by which enterocin AS-48 interacts with bacterial spores is unknown. It was observed that spores of *B. cereus* became more sensitive to enterocin AS-48 gradually after germination and were sensitive to 25–50 μg/ml 10 min after germination initiation. The greatest effect of enterocin AS-48 was observed 90–120 min after germination initiation, when cellular growth occurred (Abriouel et al., 2002). Enterocin AS-48 has also been shown to be effective in inhibiting spore outgrowth using heat activated spores of *B. cereus*. In a boiled rice substrate, 25 μg/ml of enterocin AS-48 reduced heat activated spores incubated at 37 and 15°C, below the level of detections after 3 days, whereas at 6°C, this reduction took 14 days. A higher concentration of 35 μg/ml of enterocin AS-48, reduced the heat activated spores below the level of detection in rice gruel after 24 h at three different temperatures (6, 15, and 37°C; Grande et al., 2006b).

Outgrowth inhibition of the important thermophilic spore-former *Geobacillus stearothermophilus* has also been shown using enterocin AS-48. *G. stearothermophilus* is regularly identified as a spoilage agent in low acid canned food, being highly heat resistant with a D_121°_C value of 1 min, so its removal from canned products require an extensive heat treatment (Durand et al., 2015). Viedma et al. (2009) tested the efficacy of enterocin AS-48 in inhibiting spore outgrowth of *G. stearothermophilus* using three food models, canned corn, canned peas and coconut milk, using a cocktail of two *G. stearothermophilus* strains. Here it was shown that AS-48, used at 1.75 μg/ml, reduced the viable counts of heat treated spores below the level of detection after 24 h. *B. licheniformis* was controlled in a commercial cider by AS-48 at a level of 5 μg/ml at 30°C. A significant reduction was observed in a population of germinated spores following treatment with AS-48 (Grande et al., 2006a).

The genus *Alicyclobacillus* has in recent years become a problem in the food industry. Members of this genus have an ability to grow at high temperatures (50–70°C), and at low pH values (3.0–3.5), which makes their eradication from certain foods problematic. *A. acidoterrestris* is a particular problem in acidic juice products such as apple, tomato and orange, amongst others (Steyn et al., 2011). Inhibition of *A. acidoterrestris* spores by enterocin AS-48 has been observed at concentrations as low as 2.5 μg/ml. At this concentration a reduction of 6 Log_10_ spores/ml was achieved. Using electron microscopy it was observed that the enterocin AS-48 treated spore structures sustained substantial damage supporting the hypothesis that the bacteriocin adsorbs to the spores negatively charged surface groups. This interaction with *A. acidoterrestris* would suggest a sporicidal rather than the sporostatic mechanism of action that is suggested for *B. cereus* (Grande et al., 2005).

### Lacticin 3147

Lacticin 3147, produced by *L. lactis* subsp. *lactis* DPC3147, has been shown to inhibit spores of *C. tyrobutyricum* in milk. This *Clostridium* species is responsible for late blowing in hard cheese, as their spores can survive heat treatments and germinate in the ripening cheese. Previously nitrate was used to combat clostridia but has been banned by the European Food Safety authority (EFSA) in an effort to reduce nitrosamines in food (Bassi et al., 2015). When used at a concentration of 45 μg/ml, lacticin 3147 was also able to completely inactivate 4–5 Log_10_ spores/ml over a 24 h period. Additionally, when lacticin 3147 was added following a 24 h incubation of the spores, total inactivation 6 days post addition of the bacteriocin was observed. *In situ* production of lacticin 3147, in a model curd system, has also been shown to significantly reduce (3 Log_10_ spores/g) the number of *Clostridium* spores after 13 days, when compared to a non-bacteriocin producing control. After 60 days of ripening, lacticin 3147 produced *in situ* was shown to be effective in reducing the levels of artificially contaminated clostridia (introduced prior to ripening) from 8 to 2 Log_10_ spores/g (Carmen Martínez-Cuesta et al., 2010).

### Bificin C6165

Bificin C6165 produced by *Bifidobacterium animalis* subsp. *animalis* CICC 6165 was shown to inhibit species such as *Lactobacillus, Bifidobacterium, Enterococcus, Staphylococcus*, and *Alicyclobacillus acidoterrestris*. Indeed, from an anti-sporeformer perspective, it is notable that bificin C6165 inhibited 20/20 strains of *A. acidoterrestris* tested. Bificin C6165 could also reduce a population of *A. acidoterrestris* spores and was more effective as the concentration of the bacteriocin increased (Pei et al., 2013). Another important characteristic of bificin C6165 which makes it an ideal candidate for inhibition of *A. acidoterrestris* is its activity at acidic pH 3.5–6.5 (Pei et al., 2014).

### Plantaricin TF711

Plantaricin TF711, produced by *Lactobacillus plantarum* TF711, is active over a broad pH range and is active against vegetative cells of *B. cereus* and *C. sporogenes* (Hernández et al., 2005). *C. sporogenes* acts as a research surrogate for proteolytic *C. botulinum* as these two species are closely related. This species has also been associated with late blowing of hard cheese (Bassi et al., 2015). Plantaricin TF711 was shown to reduce *C. sporogenes* spore counts significantly from 7 days onwards when introduced in the form of an adjunct culture producing the bacteriocin *in situ*. The bacteriocin was shown to be present at highest levels at day 21, after which its activity declined. This decline in activity could be due to loss of stability, depletion of the bacteriocin in the cheese, or reduced production of the bacteriocin (González and Zárate, 2015).

### Thurincin H

Thurincin H produced by *B. thuringiensis* SF361 has been shown to be sporostatic against dormant *B. cereus* spores and sporicidal against germinated *B. cereus* spores. Similarly to other bacteriocins, thurincin H displays sporicidal activity after germination, while it was sporostatic to dormant spores. Although not an LAB bacteriocin, it has been suggested that Thurincin H may have potential for use in food (Wang et al., 2014).

### Other bacteriocins active against bacterial spores

There are a number of other bacteriocins that have shown potential. Some of these are described here. Soria and Audisio (2014) revealed that heat activated *B. cereus* spores could be inhibited by the cell free supernatant of *E. faecium* SM21 containing an enterocin which produced a bacteriostatic effect at both pH 5 and pH 6. Bacteriocin production by *Streptococcus thermophilus* 580 was capable of inhibiting *C. tyrobutyricum* gas production in a ripening curd model for up to 14 days, when compared to controls which produced gas after 14 days (Mathot et al., 2003). Pentocin L and pentocin S, are produced by *Pediococcus pentosaceus* L and S, respectively. Both of these bacteriocins are inhibitory against a variety of vegetative *Bacillus* and *Clostridium* strains (Table 1). Furthermore, these bacteriocins were shown to be sporostatic by inhibiting the germination of three different strains of non-heat activated *B. cereus* spores. These active proteins are larger than typical bacteriocins which suggests that these peptides may in fact be bacteriolysins (Yin et al., 2003).

### Comparing the sensitivity of spores and vegetative cells to bacteriocins

To date there has been conflicting reports as to whether germinated spores are more or less resistant to bacteriocins than vegetative cells. Heat activated spores of *B. sporothermodurans* are less sensitive to nisin (1.25 μg/ml), than vegetative cells of *B. sporothermodurans* (Aouadhi et al., 2015). The Minimum Inhibitory Concentration (MIC) of nisin for vegetative cells of *C. butyricum, C. perfringens, C. sporogenes*, and *C. tyrobutyricum* was found to be 0.17, 0.75, 38.4, and 4.8 μg/ml, respectively. However, 23 μg/ml of nisin prevented outgrowth of heat activated *Clostridium* spores for up to 10 days. Unfortunately in this study it is unclear whether the vegetative cells were more or less resistant than their spores to the nisin treatment as no MIC for spores was carried out (Meghrous et al., 1999). Another study found that vegetative cells of *C. sporogenes* were less resistant to nisin than heat activated spores, yielding MICs of 0.23 and 1.11 μg/ml, respectively. In contrast, it was revealed that heat activated *C. beijerinckii* spores were less resistant with an MIC of 1.09 μg/ml while their vegetative cells exhibited an MIC of 1.3 μg/ml (Hofstetter et al., 2013). At odds with these findings, however, were the results obtained by Ávila et al. (2014), which compared the sensitivity of spores and vegetative cells of four clostridia: *C. tyrobutyricum, C. butyricum, C. beijerinckii*, and *C. sporogenes*. Using four representatives of each species, they showed that spores had a higher MIC, and thus were more resistant to nisin, than their vegetative counterparts in 15 of the 16 strains tested. The only exception was displayed by *C. tyrobutyricum* CET 4011 strain where the vegetative and spore MIC values were equal at 0.39 μg/ml. It is also important to note that in this case all the MIC values were below the maximum permissible limit for nisin, which is 12.5 μg/ml in Europe.

Spores of *A. acidoterrestris* were found to be more sensitive to nisin than their vegetative cells. The MIC values for both spores and vegetative cells were carried out in mYPGA at two different pH values (pH 3.4 and pH 4.2). Interestingly, at pH 3.4, all spores were more sensitive (7/7) than their vegetative cells. However, at pH 4.2 (3/7) spores had equal MIC-values to their vegetative cells (Yamazaki et al., 2000). Whether this is due to the (i) enhanced activity of nisin at lower pH, (ii) negative effects of pH on the spore or (iii) a combined activity of both, has yet to be determined. These findings were further confirmed by Ruiz et al. (2013), who found the MIC of spores and vegetative cells of *A. acidoterrestris* to be 7.81 and 31.25 μg/ml, respectively.

### Inhibition of spore outgrowth prevents toxin formation

Toxin formation is an important feature of a number *Clostridium* and *Bacillus* species. There are two types of toxin with which *B. cereus* strains are frequently associated: (i) heat labile diarrheal enterotoxin and/or (ii) heat-stable emetic enterotoxin. Beuchat et al. (1997) showed that the production of diarrheal enterotoxin produced in beef gravy inoculated with *B. cereus* spores could be inhibited by addition of nisin. Enterotoxin production normally occurred after 3 and 9 days for heat activated *B. cereus* spores incubated at 15 and 8°C, respectively. Addition of 1 μg/ml of nisin inhibited enterotoxin production completely at 8°C, whereas a higher concentration of 5 μg/ml was needed to inhibit enterotoxin production at 15°C over a 14 day period. The levels of nisin required to prevent enterotoxin production from a spore inoculum also ensured that the final cell numbers did not exceed 4.03 and 6.23 Log_10_ CFU/g at 8 and 15°C, respectively. Without nisin, enterotoxin was produced when cell numbers exceeded 6.78 and 7.1 Log_10_ CFU/ml at 15 and 8°C, respectively. This is in agreement with the strategy of keeping the *B. cereus* population below ~7 Log_10_ CFU/g to prevent enterotoxin production (Christiansson et al., 1989). It would be interesting to see if the cell numbers in the presence of nisin were allowed to exceed these numbers would enterotoxin be still be produced or would the enterotoxin production cease due to the presence of nisin.

Enterocin AS-48 was also shown to have an effect on enterotoxin production by psychrotrophic vegetative cells of *B. cereus*. Enterocin AS-48 completely inhibited enterotoxin production and bacterial growth for at least 72 h when used at 7.5 μg/ml. When enterocin AS-48 was used at subinhibitory concentrations (2.5 or 5 μg/ml) the growth of the cells were severely subdued and enterotoxin titres were 10-fold lower than non-bacteriocin treated controls (Abriouel et al., 2002).

## Combining bacteriocins with other hurdles

### Bacteriocins in combination with heating

The thermal resistance of bacterial spores makes their eradication from food by heat a major problem during food processing. Nisin at various concentrations has been shown to reduce the decimal reduction times (*D*-values) and thus the thermal resistance of bacterial spores. Therefore, nisin has been described as a compound with a “two-fold beneficial effect”: (i) it enhances the heat sensitivity of the bacterial spore (Table 4) and (ii) it prevents the outgrowth of spores which survive the heat treatment (Komitopoulou et al., 1999). Pre-exposing heat activated *G. stearothermophilus* spores to nisin (50 μg/ml) at 4°C in chocolate milk for 15 and 24 h, resulted in significantly reduced *D*_130°_C values of 20.5 and 25.1%, respectively, compared to those spores not pretreated with nisin. When the nisin pretreatment was raised to 100 μg/ml this did not cause a significant reduction over the lower concentration of 50 μg/ml (Beard et al., 1999). *B. amyloliquefaciens* spores were rapidly inactivated when treated with 90°C and 16 μg/ml of nisin, in contrast to the results when a 90°C treatment was used, alone, where there was no inactivation of spores (Hofstetter et al., 2013).

**Table 4:** **Nisin addition to food reduces spore *D*-values**.

**Spore**	**Nisin Conc μg/ml**	**Food model**	**D_x°C_**	**% decrease in *D*-value compared to non-nisin treated control**	**References**
*B. cereus*	25	Milk	*D*_80°C_	40	Vessoni and Moraes, 2002
			*D*_90°C_	16	
			*D*_97.8°C_	46	
*B. cereus*	50	Milk	*D*_97°C_	32	Wandling et al., 1999
			*D*_100°C_	20	
			*D*_103°C_	42	
*G. stearothermophilus*	100	Milk	*D*_130°C_	20	Wandling et al., 1999
*A. acidoterrestris*	1.25	Apple juice	*D*_80°C_	42	Komitopoulou et al., 1999

A reduction of 2 Log_10_ spores/ml was observed when spores of *C. sporogenes* spores were subjected to a heat treatment of 90°C for 2 h in the presence of 16 μg/ml nisin vs. a 90°C heat treatment without nisin. Additionally there was 30% greater DPA release when spores of *C. sporogenes* were heat treated at 90°C with nisin than those spores which were not treated in any way. However, when *C. beijerinckii* was subjected to the same conditions (90°C for 2 h and 16 μg/ml nisin), no increased inactivation was observed. The ability of nisin to increase the permeability of resting spores of *C. sporogenes* and *C. beijerinckii* was observed using DAPI staining. Fluorescence was observed after a treatment at 90°C with nisin, whereas a heat treated spore without nisin that did not fluoresce (Hofstetter et al., 2013). These findings are consistent with the hypothesis that nisin lowers the heat resistance of spores by permeabilizing their exterior.

Response surface technology (RSM) is an empirical modeling technique that can be used to examine and predict the relationship between the response variable and the test variable. RSM can be used to predict optimum processing conditions to achieve a pre-determined reduction in spores (Table 5).

**Table 5:** **RSM models can be used to predict a treatment to achieve a specific spore reduction in food**.

**Spore**	**Predicted reduction**	**Treatment**				**Food model employed**	**References**
		**Pressure (MPa)**	**Temperature (°C)**	**Time (min)**	**Nisin (μg/ml)**		
*B. sporothermodurans*	5 Log_10_ spore/ml		95	12	3.125	Water	Aouadhi et al., 2014
*B. sporothermodurans*	5 Log_10_ spore/ml		100	13	3.35	Milk	
*B. sporothermodurans*	5 Log_10_ spore/ml		100	15	3.375	Chocolate milk	
*B. sporothermodurans*	5 Log_10_ spore/ml	472	53	5	5.025	Water	Aouadhi et al., 2013
*C. perfringens*	6 Log_10_ spore/ml	654	74	13.6	8.2	UHT milk	Gao et al., 2011

Dormant *B. coagulans* spores were shown to be resistant to enterocin AS-48 in that use of 6 μg/ml bacteriocin resulted in an approximately one log reduction in the number of viable cells when dormant spores were treated with the bacteriocin in three food models: (i) tomato paste, (ii) syrup from canned peach, and (iii) juice from canned pineapple. However, using enterocin AS-48 at 3 and 6 μg/ml in combination with heat treatments (5 min at a minimum of 80°C) showed a significant reduction in the number of viable cells in both food models. When spores were incubated at 22°C for 48 h with bacteriocin, then heat treated at both 80 and 95°C, there was a significant difference in the number of viable cells obtained following both treatments relative to the non-heat treated controls or those that were heat treated without bacteriocin (Lucas et al., 2006). A relationship between heat temperature and survivors was observed, showing that viable counts in samples supplemented with bacteriocin decreased as the temperature was increased. This relationship was further evidenced by the significant reduction in viable counts obtained from bacteriocin treated spores heat treated at 95°C over those heat treated at 80°C. This relationship was observed in all three food models previously discussed (Lucas et al., 2006). Ultimately, this study nicely highlights the efficacy of bacteriocins to (i) reduce the severity of heat treatments and (ii) increase the effectiveness of heat treatments, when used to inactivate spores in food.

Another bacteriocin discussed previously, bificin C6165, has been shown to reduce the *D*_90°C_ value of *A. acidoterrestris* as the bacteriocin concentration increased from 0 to 160 μg/ml. Addition of 80 and 160 μg/ml of bificin C6165 was shown to reduce the *D*_90°C_ *A. acidoterrestris* CFD1 by 32.7 and 42.7%, respectively (Pei et al., 2014).

### Bacteriocins in combination with high pressure

High-pressure processing (HPP) is a “non-thermal” food preservation technique that inactivates harmful pathogens and vegetative spoilage microorganisms by using pressure rather than heat to effect pasteurization. HPP utilizes intense pressure (about 400–600 MPa or 58,000–87,000 psi) at chilled or mild process temperatures (< 45°C), allowing most foods to be preserved with minimal effects on taste, texture, appearance, or nutritional value. Microorganisms do however display a variability in their sensitivity to HHP in the order: Gram-negative bacteria > Gram-positive bacteria > bacterial spores. While HPP is an effective method used for the destruction of microorganisms in food, it is not sufficient alone to inactivate spores and therefore must be combined with other hurdles, such as bacteriocins, to increase its efficacy. Indeed, treating food with bacteriocins may be an excellent combination as HHP can induce germination, which can facilitate the germination-dependent sporicidal activity of bacteriocins. Black et al. (2008) showed that treatment of 8 Log_10_ spores/ml of *B. subtilis* with low pressure (100 MPa i.e., not HHP) at 40°C in milk resulted in germination and inactivation of 4 and 1 Log_10_ spores/ml, respectively. A similar level of germination, but without inactivation, was observed in milk when a higher treatment of 500 MPa was used. When spores were treated with a combination of HP (500 MPa) and nisin (12.5 μg/ml), spore germination and inactivation increased to 6 and 3 Log_10_ spores/ml, respectively. When cycled twice with nisin there was a further increase in spore germination and inactivation of 8 and 6 Log_10_ spores/ml, respectively. High pressure-induced germination is known not to require the presence of nutrient receptors and is characterized by a rapid release in DPA-Ca^2+^ from the core. Nisin can be characterized as a potent pro-germinant in the presence of germinants (naturally present in milk) such as L-alanine and L-cysteine. Interestingly, nisin doubled the rate of germination in *C. botulinum* spores, while it had no effect on nisin resistant spores (Mazzotta and Montville, 1999). It was hypothesized that inactivation of spores by HHP and nisin could be due to (i) nisin and HP acting synergistically to inactivate spores or (ii) HP inducing germination after which nisin exerts its lethal effect on the germinated spore (Black et al., 2008). *C. sporogenes* spores were also shown to be inhibited rapidly by a treatment of nisin and 600 MPa at 90°C, relative to a treatment of 90°C alone (Hofstetter et al., 2013).

More recently, several studies have used response surface methodology (RSM) to test the effectiveness of high pressure, heat and nisin. Aouadhi et al. (2013) used RSM to investigate the effects of high pressure, in combination with moderate heat and nisin treatment, on *B. sporothermodurans* spores. The authors showed that spore inactivation was concentration dependent and that 1.25 and 125 μg/ml caused an inactivation of 0.4 and 4 Log_10_ spores/ml, respectively. Aouadhi et al. (2014) and Gao et al. (2011) showed that RSM (Table 5) could be effectively implemented to design an optimum treatment, involving multiple parameters to reduce spores loads by a predetermined amount.

Interestingly, superdormant spores of *B. cereus* and *B. subtilis* have been shown to germinate similarly to dormant spores when treated with pressure of 150MPa regardless of whether they were heat-activated or non-heat-activated. There have, however, been conflicting reports regarding the ability of pressure treatments to cause germination of an entire spore population. This uncertainty has impeded the widespread use of high pressure. It has been hypothesized that spores which remain superdormant after high pressure may do so via a distinct mechanism from that involved in making some spores superdormant to nutrient germinants (Wei et al., 2010).

### Bacteriocin in combination with pulsed electric field

Pulsed electric field (PEF) is an innovative food preservation method, which may be suitable for reducing spore loads in liquid food. One of the distinct advantages of PEF is that the thermal impacts on food are minimized as this treatment is relatively non-thermal. Any heat produced is directly influenced by the energy input of the treatment. While it is known that vegetative cells of *B. cereus* are sensitive to PEF and nisin (Pol et al., 2000) and that this combination is sporostatic but this treatment did not initiate germination nor did they affect the viability of the dormant spores. After germination, *B. cereus* immediately became sensitive to nisin (1.25 μg/ml) but it was longer (50 min) before they became sensitive to PEF (27 kV/cm, 302-μs pulses; flow rate, 10 ml/min). Unlike the synergistic activity of nisin and PEF against vegetative cells (Pol et al., 2000), when spores when treated with both PEF and nisin this synergistic activity was not observed as the reduction was comparable to nisin alone (Pol et al., 2001). While this combination is not synergistic against spores, food rarely contains spores alone but rather a mixed population of spores and vegetative cells. Therefore, this combination may still be an effective way of maintaining dormant spore numbers yet reducing the population of vegetative cells for increased food safety and shelf life.

### Bacteriocins in combination with osmotic activation

Stimulation of dormant bacterial spore germination followed by subsequent inactivation, as previously discussed, is a promising method used for spore inactivation. Small, non-polar, hydrophobic solutes that permeate the plasma membrane have been shown to stimulate *B. cereus* germination (Preston and Douthit, 1984). Inhibition of non-heat activated *C. difficile* spores was significantly increased when treated with nisin and single osmotic activators (ammonium, glycerol, and Tris) compared to heat activated spores treated with nisin and solutes in a germination medium. For example, nisin in combination with heating resulted in a 1–2.5 log_10_ spores/ml decrease in viable spores but when nisin was combined with osmotic activators this increased to >3.5 log_10_ spores/ml (Nerandzic and Donskey, 2013). Using flow cytometry, it was observed that the membrane permeability of spores was significantly increased when treated with osmotic activators. Spores treated with both nisin and solute transitioned to phase dark (as spores germinate they appear phase dark using phase contrast microscopy), whereas those incubated with nisin and osmotic activators separately did not transition to phase dark (Nerandzic and Donskey, 2013). The proposed synergistic ability of nisin and osmotic activators to inhibit outgrowth was attributed to the osmotically induced loss of membrane integrity. Although *C. difficile* is of clinical importance, this use of osmotic activation could be used to overcome limitations of the germination dependent activity of bacteriocins with other food related strains of clostridia.

### Bacteriocins in food packaging

The preservation of sausage casings of preserved intestines of animals has been practiced for centuries. However, this preservation method has been modernized to suit modern consumer desires. Such a modernization is the binding of nisin to sausage casing in order to control *Clostridium* spore outgrowth. Wijnker et al. (2011) showed that nisin, at 100 μg/ml, when bound to casings and placed on agar plates seeded with *Clostridium* spores, produced zones whereas those casings with only 50 μg/ml did not. They also observed that addition of nisin at 50 μg/ml to the casings delayed *C. sporogenes* spore outgrowth between 1 and 8 days. Furthermore, at this concentration of 50 μg/ml, this sporostatic activity was observed for 30 days. In contrast, Meghrous et al. (1999) showed that a lower concentration of nisin, 23 μg/ml, delayed *clostridial* spore outgrowth by 10 days. It should also be noted that Wijnker et al. (2011) used 10^6^ spores/ml whereas Meghrous et al. (1999) used 10^3^ spores/ml. The reason that nisin at 50 μg/ml could inhibit outgrowth *in vitro* but not on the casings could be due to the irreversible binding of nisin to the collagen matrix of the casing wall. This would suggest that if outgrowth is to be prevented the casings need to contain a higher concentration of nisin in order to overcome the deleterious effect of irreversible binding to the casing.

### Bacteriocins in combination with plant extracts

Plants contain innumerable constituents and are valuable sources of new and biologically active molecules possessing antimicrobial properties. The plant family *Piperaceae* are found in tropical and subtropical regions and are commonly used as to generate medicinal herbs. Ruiz et al. (2013) showed that a combination of nisin and *Piper aduncum* exhibited a strong antibacterial activity against spores of *A. acidoterrestris* and also exhibited a synergism (FIC = 0.24) against *A. acidoterrestris* vegetative cells. Prenylated chromone was identified as the active compound in this plant extract. *Piperaceae* extract is a natural food preservation method that may be combined with nisin to lower (if any) heat treatment needed to reduce and inhibit spores outgrowth.

### Nisin in combination with fatty acid esters

Sucrose fatty esters are approved internationally for use as emulsifiers and these non-toxic molecules have also been reported to inhibit Gram-positive bacteria. A combination of nisin and the fatty acid ester, sucrose palmitate (P-1570), displayed synergism against spores of *B. cereus* whereas sucrose fatty acid esters alone caused no decrease in growth (Thomas et al., 1998). Total inhibition of *B. licheniformis* spore outgrowth was achieved when nisin (0.75 μg/ml) was combined with the fatty acid ester monolaurin (100 μg/ml) whereas when these treatments were used separately at higher concentrations they only partially inhibited outgrowth (Mansour et al., 1999).

### Nisin in combination with potassium sorbate

Sorbates are extensively used in the food industry, as they are able to inhibit, or delay growth of, spores and vegetative populations of bacteria. Although their mechanism of action is not full defined for bacterial spores, it is has been shown that potassium sorbate inhibits the growth of spores of *Bacillus* species (Oloyede and Scholefield, 1994). A combination of nisin (1.25 μg/ml) and potassium sorbate (2% w/v) has been shown to cause a synergistic reduction in the number of heat activated *B. sporothermodurans* spores. After 8 h there was ~3 Log_10_ spores/ml reduction in spores. This reduction in spores continued albeit at a slower rate until 5 days where total inhibition of *B. sporothermodurans* spores occurred (Aouadhi et al., 2015). When tested separately at these levels, both nisin and potassium sorbate inhibited spore outgrowth. Nisin was not sporicidal but rather sporostatic, inhibiting spore outgrowth. While potassium sorbate was not sporicidal, it did significantly perturb germination of *B. sporothermodurans* and inhibited the outgrowth of spores (Aouadhi et al., 2015). This ability of potassium sorbate to inhibit spore germination has previously been reported for spores of *B. cereus* and *C. botulinum* (Smoot and Pierson, 1981).

## Discussion

While spores are a widely recognized problem in the food industry the majority of bacteriocin-related studies have focused on the elimination of vegetative cells from food. The removal of spores and inhibition of their outgrowth in food is important for (i) increasing shelf life and (ii) protecting the consumer from harmful pathogenic spore-formers. Although, there are numerous bacteriocins which have been characterized as safe and effective molecules for use in food, to date, nisin is the only bacteriocin which is authorized for use as a food preservative. While this bacteriocin provides an effective and safe method to reduce spore outgrowth in food, it is important to recognize that this molecule has its limitations. Bacteriocins in food may be limited by: molecule specific solubility, the active pH range of the bacteriocin, inactivation by proteases in food, and the possible negative interactions that occur between certain bacteriocins and certain food components. One such limitation of nisin is its loss of activity as the pH of the food increases. There are a variety of bacteriocins which are more active than nisin at higher pH, such as gassericin A, pediocin AcM, and thermophilin T (Table 1), however they still need to be further characterized before their use in food may be authorized.

In the majority of cases nisin is only sporicidal against those spores in the outgrowth phase and therefore has no effect on those spores in the dormant phase. Although this model of nisin (and other bacteriocins) use in food suggests that germination is a prerequisite for its activity, it is important to note that there are relatively few studies which investigate bacteriocin/spore interactions. Furthermore, it should be recognized that the only detailed mechanism for bacteriocins/spore interaction is that of *B. anthracis* (**Figure 2**). Indeed, the limited number of existing studies highlights the need for further research in this area. Understanding these interactions and mechanisms will ultimately lead to a more precise and optimal use of bacteriocins in food. Undeniably, the mode of action for a great many bacteriocins has yet to be elucidated and a better understanding of the methods by which bacteriocins kill bacteria will facilitate a solid basis for engineering new and more potent derivatives with optimized potency and stability. Given that spores must germinate to exert their adverse effects, future research should focus on stimulating spore germination to enable spores to be more effectively targeted by bacteriocins in food settings. Indeed, recent research provides stimulating evidence for using a germination step prior to spore destruction for promoting inactivation of *Bacillus* and *Clostridial* spores (Gut et al., 2008). Furthermore, although numerous components of the spore germination machinery are conserved between spore forming members of bacilli and clostridia, significant differences between the germination of spores of *Clostridium perfringens* and that of spores of a number of *Bacillus* species, both in the proteins and in the signal transduction pathways involved have been revealed (Abhyankar et al., 2014; Setlow, 2014a; Olguín-Araneda et al., 2015). Indeed, as the number of microbial genome sequences has increased dramatically, bioinformatics data contained in the large number of spore-forming Bacillales and Clostridiales genomes that have been sequenced and the information gained from their analysis, can be used to guide researchers to develop novel strategies to achieve a complete and permanent loss of the spore's ability to germinate and grow in food products.

Regardless of the specific bacteriocin of choice, it is clear that there is considerable evidence of the potential value of bacteriocins with respect to controlling sporeforming bacteria in food. In the case of spores, while this activity more frequently tends to be sporostatic, there are also examples of sporicidal effects. As is the case for vegetative cells, the mechanisms via which bacteriocins inhibit spores may be heterogeneous but ultimately it is apparent that in general bacterial spores can be controlled using bacteriocins, and their application in combination with other novel non-thermal treatments makes their efficacy even greater. The use of the bacteriocins with other food processing hurdles, such as those previously described, thus has the potential to satisfy consumer demands for “clean label” products, enabling processors to produce foods of optimal quality and shelf life.

## Author contributions

KE drafted the manuscript. DF, MR, RR, CH, and PC revised and approved the final manuscript.

## Funding

KE, DF, CH, PC, MR, RR are supported by the Irish Government under the National Development Plan, through the Food Institutional Research Measure, administered by the Department of Agriculture, Fisheries and Food, Ireland (DAFM 13/F/462) to PC and MR, a Science Foundation Ireland (SFI) Technology and Innovation Development Award (TIDA 14/TIDA/2286) to DF, SFI-PI funding (11/PI/1137) to PDC and the APC Microbiome Insitute under Grant Number SFI/12/RC/2273.

### Conflict of interest statement

The authors declare that the research was conducted in the absence of any commercial or financial relationships that could be construed as a potential conflict of interest.
